# Twist: A Regulator of Epithelial-Mesenchymal Transition in Lung Fibrosis

**DOI:** 10.1371/journal.pone.0007559

**Published:** 2009-10-23

**Authors:** Veronika Pozharskaya, Edilson Torres-González, Mauricio Rojas, Anthony Gal, Minal Amin, Sheila Dollard, Jesse Roman, Arlene A. Stecenko, Ana L. Mora

**Affiliations:** 1 Division of Pulmonary, Allergy/Immunology, Cystic Fibrosis and Sleep, Department of Pediatrics, Emory University, Atlanta, Georgia, United States of America; 2 CTRL, Division of Pulmonary, Allergy and Critical Care, Department of Medicine, Emory University, Atlanta, Georgia, United States of America; 3 McKelvey Lung Transplantation Center, Emory University, Atlanta, Georgia, United States of America; 4 Department of Pathology, Emory University, Atlanta, Georgia, United States of America; 5 National Center for Immunization and Respiratory Diseases, CDC, Atlanta, Georgia, United States of America; 6 Department of Medicine, University of Louisville School of Medicine and Louisville VA Medical Center, Louisville, Kentucky, United States of America; Helmholtz Zentrum München/Ludwig-Maximilians-University Munich, Germany

## Abstract

**Background:**

Several studies have implicated viral infection as an important factor in the pathogenesis of IPF and related fibrotic lung disorders. Viruses are thought to cause epithelial cell injury and promote epithelial-mesenchymal transition (EMT), a process whereby differentiated epithelial cells undergo transition to a mesenchymal phenotype, and considered a source of fibroblasts in the setting of lung injury. We have demonstrated an association between the epithelial injury caused by chronic herpes virus infection with the murine γ-herpes virus, MHV68, and lung fibrosis. We hypothesize that EMT in this model of virus-induced pulmonary fibrosis is driven by the expression of the transcription factor Twist.

**Methods/Findings:**

*In vitro* MHV68 infection of murine lung epithelial cells induced expression of Twist, and mesenchymal markers. Stable overexpression of Twist promoted EMT in MLE15 lung epithelial cells. Transient knockdown expression of Twist resulted in preservation of epithelial phenotype after *in vitro* MHV68 infection. In concordance, high expression of Twist was found in lung epithelial cells of MHV68 infected mice, but not in mock infected mice. Alveolar epithelial cells from lung tissue of idiopathic pulmonary fibrosis (IPF) patients were strongly positive for Twist. These cells demonstrated features of EMT with low expression of E-cadherin and upregulation of the mesenchymal marker N-cadherin. Finally, IPF tissue with high Twist protein levels was also positive for the herpesvirus, EBV.

**Conclusions/Significance:**

We conclude that Twist contributes to EMT in the model of virus-induced pulmonary fibrosis. We speculate that in some IPF cases, γ-herpes virus infection with EBV might be a source of injury precipitating EMT through the expression of Twist.

## Introduction

Current understanding of the pathogenesis of Idiopathic Pulmonary Fibrosis (IPF) considers ongoing injury of the lung epithelium as one of the most important drivers of the disease. The cause of this injury remains unknown although chronic herpesvirus infection has been suggested by several studies that show the presence of one or more herpes viruses in the lungs of IPF patients [1], [2], [3], [4], [5], [6]. Both Epstein-Barr virus (EBV) protein and DNA expression have been identified in lung tissue of patients with IPF. Evidence that viral infection may be causally related to the development of IPF is the observation that expression of EBV latent membrane protein 1 (LMP-1) in alveolar cells is associated with a poor prognosis in IPF patients, and a major host cell for the virus is the alveolar epithelial cell [7]. In addition, we have demonstrated that Interferon-γ-Receptor knockout (IFNγR−/−) mice inoculated with the murine γ-herpes virus 68 (MHV68) develop progressive interstitial fibrosis, and many histological features reminiscent of IPF associated with virus reactivation [8]. Furthermore, using the same murine model, we have shown that antiviral therapy controls virus replication and prevents lung fibrosis [9].

Recently, we turned our attention to the factors that control fibroblast proliferation in this model of virus-induced lung fibrosis. Fibroblasts from fibrotic lungs can originate locally by proliferation of resident interstitial fibroblasts. They can also originate from circulating fibrocytes derived from bone marrow progenitor cells or from the transdifferentiation of epithelial cells [10], [11]. In the latter, the epithelial cells undergo morphological changes to acquire fibroblast or myofibroblast markers such as fibroblast-specific protein 1 (FSP-1), α-smooth muscle actin, N-cadherin, and vimentin, while losing epithelial markers such as E-cadherin, occludin, and desmoplakin. This process is termed epithelial-mesenchymal transition (EMT). Histopathology analyses of IPF lung biopsies and animal models of pulmonary fibrosis have revealed epithelial cells which acquire a mesenchymal phenotype giving evidence to the idea that EMT may be an important source of fibroblasts in the setting of IPF [12], [13].

We set out to investigate the role of EMT in virus-induced lung fibrosis and the factors that influence this process. A crucial role has been identified for TGF-β in the induction of EMT[14]. Primary alveolar epithelial cells in culture exposed to TGF-β for 3–6 days undergo transdifferentiation to fibroblasts and myofibroblasts [12]. TGF-β expression is upregulated at the sites of the epithelial injury in IPF, thus it is likely a factor involved in EMT. Another driver of EMT appears to be Twist protein as demonstrated in renal fibrosis [15] and various tumors [16]. Twist encodes a basic helix-loop-helix transcription factor required for EMT during embryogenesis and carcinogenesis [17]. Homozygous mutant Twist-null embryos die at 11.5 days with an obvious failure of neural tube closure [18]. Hypoxia, epidermal growth factor receptor, nuclear factor κB (NF-κB), TGF-β and Wnt1 are known inducers of Twist [19], [20], [21], [22], [23]. In the human nasopharyngeal carcinoma associated with EBV infection, Twist expression has been found to be induced by EBV protein LMP-1 via NF-κB [24].

The mechanisms responsible for fibroblast generation in the setting of virus-induced lung fibrosis are unknown. We believe that EMT is a key event in this setting and hypothesize a role for Twist in this process. In this study, we show evidence that Twist might indeed play a role in EMT and that γ-herpes viruses contribute to EMT by upregulation of Twist.

## Results

### Twist expression and EMT in cultured alveolar epithelial cells

**Figure 1:**
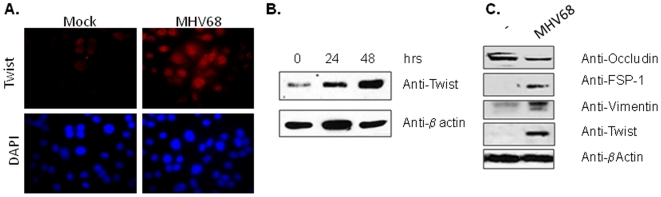
Expression of Twist is induced by *in vitro* infection of lung epithelial cells with MHV68. MLE-15 cells were mock-infected or infected at multiplicity of infection of 0.01 with MHV68 and analyzed 48 h post-infection for Twist expression. (A) Immunostaining of infected cells using an anti-Twist antibody. Twist positive staining (red) was observed in nuclei of MHV68 infected cells but not in mock infected cells. Cells were counterstained with DAPI (blue). Magnification ×1000. Staining is representative of 2 independent experiments. (B) Western blot analysis for Twist in whole cell lysates. The blot was stripped and reprobed with anti-β-actin antibody as loading control. (C) Western blot analysis for Twist, epithelial (Occludin) and mesenchymal (FSP-1, Vimentin) markers in whole cell lysates from uninfected and MHV68 infected MLE-15 cells. Notice the increase in the expression of mesenchymal markers and the decrease in Occludin expression after infection.

EBV infection of nasopharyngeal epithelial cells results in EMT and malignant transformation in humans. This process is thought to be regulated by the transcription factor Twist [24]. Since MHV68 is the murine homologue of EBV, we examined whether MHV68 infection could induce Twist *in vitro*. To this end, murine lung epithelial cells, MLE-15, were infected with MHV68 or mock infected. Twist expression was detected by immunofluorescent staining in the cell nuclei two days after infection (Fig. 1A). There was no Twist staining in mock infected cells with the exception of a few cells with lower intensity cytoplasmic signal. Twist levels in infected and mock infected cells were also analyzed by Western blotting. As shown in Fig. 1B, high levels of Twist protein were observed exclusively in MHV68 infected cells at 24 and 48 h post-infection. In addition, we analyzed the expression of epithelial and mesenchymal markers after 48 h post-infection in MLE15 cells. A diminution in the epithelial marker Occludin accompanied by an increase in the mesenchymal markers Vimentin, and fibroblast-specific protein 1 (FSP1) was observed in MHV68 infected cells compared to uninfected cells (Fig. 1C).

**Figure 2:**
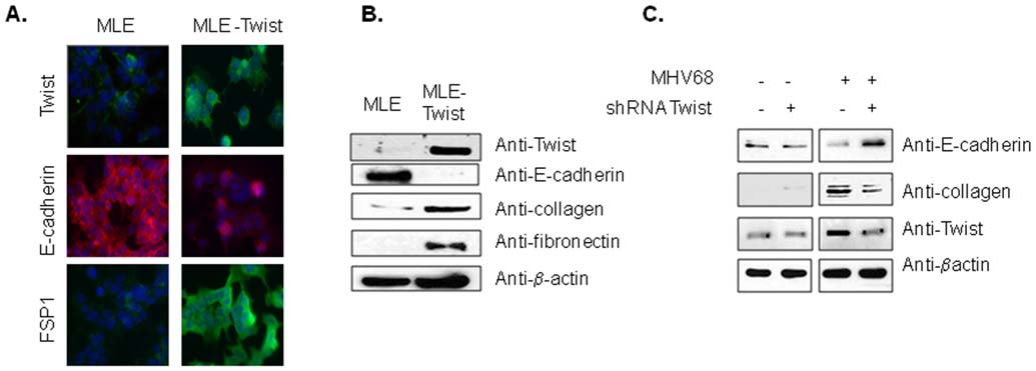
Twist promotes EMT in lung epithelial cells. (A) Stable transfection of MLE-15 cells was carried out with Twist1 cDNA. Immunofluorescent staining was performed using anti-Twist (green), anti-E-cadherin (red), and anti-FSP-1 (green) antibodies. Twist positive cells showed loss of E-cadherin staining in the cell membrane. Cells were counterstained with DAPI (blue). Magnification is ×400. Staining is representative of 3 independent experiments. (B) Western blot analysis for Twist, epithelial (E-cadherin) and mesenchymal (Collagen IV and Fibronectin) markers in whole cell lysates from nontransfected and Twist-transfected MLE-15 cells. The blot was stripped and reprobed with anti-β-actin and Twist antibodies as controls. (C) Immunoblot for Twist, E-cadherin (epithelial marker) and Collagen I (mesenchymal marker) from MLE-15 cells transduced with lentiviral constructs expressing control shRNA or Twist1 shRNA as indicated followed by MHV68 infection.

To determine whether Twist expression was able to promote EMT, we made stable transfection of MLE-15 cells using Twist cDNA and examined expression of mesenchymal and epithelial cell markers. Control cells maintained a cobblestone-like appearance with polygonal shape of cells and expressed the epithelial marker, E-cadherin, in the membrane compartment as determined by immunostaining. Twist-transfected cells lost the cobblestone appearance, became more scattered, and expressed low levels of E-cadherin in the cytoplasm (Fig. 2A). At the same time, the cytoplasm of these cells showed positive staining for a mesenchymal marker, FSP-1. Western blot analysis of whole MLE-15 cell extracts demonstrated high levels of E-cadherin in nontransfected cells. There were low levels of E-cadherin and high levels of collagen IV and fibronectin in Twist overexpressing cells (Fig. 2B). Thus, ectopic overexpression of Twist resulted in phenotypic change of MLE-15 shown by induction of mesenchymal markers including several ECM proteins and altered expression of the epithelial marker, E-cadherin.

To determine whether Twist plays a causal role in the changing epithelial phenotype after infection, we tested whether inhibition of Twist expression would affect MHV68 ability to induce EMT. MLE15 cells were transduced with lentiviral particles expressing Twist shRNA and infected with MHV68 24 h later. Forty-eight hours after MHV68 infection, whole cell lysates were prepared and analyzed by Western blot using anti-Collagen I and E-cadherin antibodies. We found that inhibition of Twist expression was accompanied by persistent E-cadherin expression and lower expression of Collagen compared to cells with knockdown expression of Twist (Fig. 2C).

### Twist is expressed in lung epithelial cells during in vivo MHV68 infection

**Figure 3:**
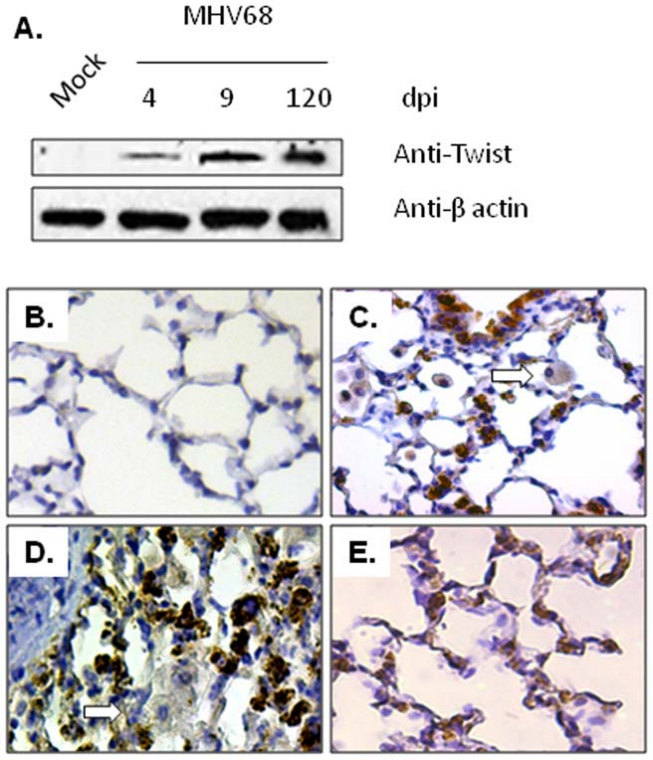
Expression of Twist is induced in alveolar epithelial cells after *in vivo* infection with MHV68. (A) Expression of Twist was analyzed by Western blot in lung homogenates from IFNγR−/− mice after mock- and MHV68-infection at days 4, 9 and 120 post-infection. Time-dependent increased levels of Twist were found in samples from virus-infected animals. Blots were stripped and reprobed with an anti-β-actin antibody as loading control. (B–D) Detection of Twist expression by IHC staining in lungs of IFNγR−/− mice mock infected (B), and virus-infected at 15 dpi (C), and at 120 dpi (D). Notice the abundant nuclear and cytoplasmic positive staining of lung epithelial cells in the infected mice. Alveolar macrophages were weakly positive for Twist staining (block arrow). (E) Twist detection by IHC staining in lung of C57BL/6 mice infected with MHV68 at day 15 post-infection. Magnification ×400. Staining is representative of 5 different mice at each time point.

Having established that MHV68 infection promotes Twist expression *in vitro*, and that Twist expression is associated with EMT, we determined whether Twist was expressed in the lungs of IFNγR−/− mice infected with MHV68. For this, whole lung protein was extracted from MHV68-infected and mock infected animals and analyzed by immunoblotting. There was no expression of Twist protein in lung lysates from mock-infected mice (Fig. 3A). In contrast, Twist was detected in murine lungs during the acute phase of infection (4–15 days post infection, dpi) and levels became even more prominent in chronically infected (90–120 dpi) murine lungs. Immunohistochemistry (IHC) revealed nuclear and cytoplasmic expression of Twist in the alveolar lining epithelial cells of lungs from infected, but not from mock infected animals (Fig. 3B–D). Bronchiolar epithelial cells were also Twist positive in acute infected lungs. There was weak cytoplasmic staining of some intraalveolar macrophages. In chronic inflammatory diseases, expression of Twist in Th1 lymphocytes has been reported to limit the expression of the IFNγ [25]. To determine if defective IFNγ signaling in IFN-γR−/− mice was associated with high levels of Twist, we analyzed expression levels of Twist by IHC at 15 days post-infection in lungs of C57BL/6 infected mice. C57BL/6 mice served as a background strain for IFN-γR−/− mice. We observed positive staining for Twist in alveolar epithelial cells that was similar to the staining seen in infected IFNγR−/− mice (Fig. 3E).

### MHV68 infected lung epithelial cells express both epithelial and mesenchymal cell markers

**Figure 4:**
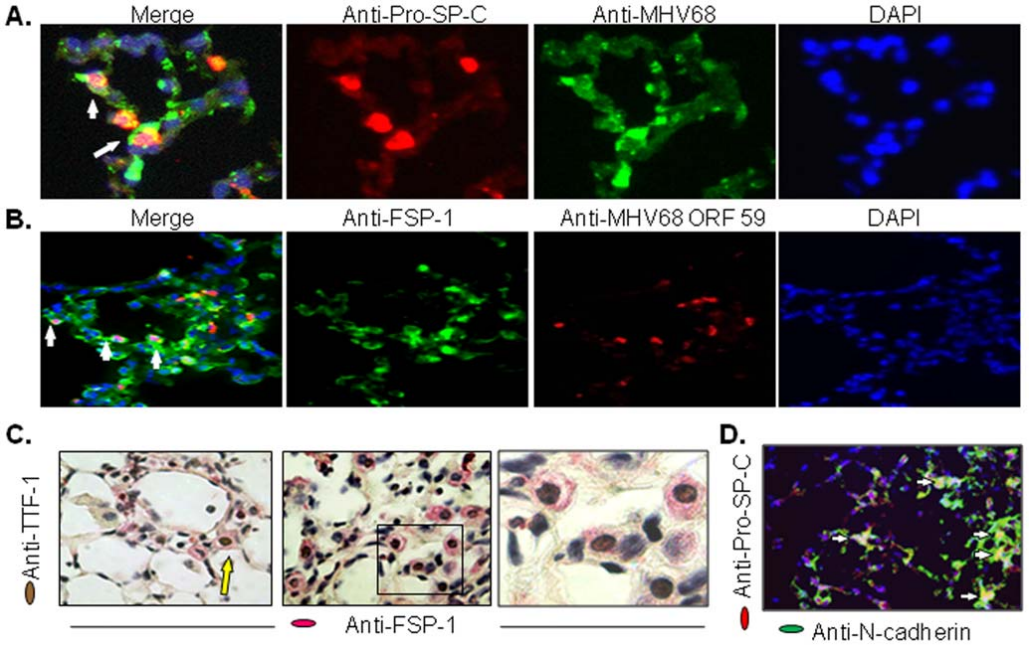
MHV68 infected lung epithelial cells express epithelial and mesenchymal cell markers *in vivo*. (A) Immunofluorescent staining of frozen lung sections of MHV68 infected IFNγR−/− mice at day 7. Virus antigen detection was performed using an anti-MHV68 polyclonal antibody (green). Type II cells were detected using anti-pro-SP-C antibody (red). Sections were counterstained with DAPI (blue). Merged image shows yellow cells indicating type II cells that support lytic infection of the virus (arrows). Magnification ×800. Staining is representative of 5 different animals. (B) Immunostaining of frozen lung sections of IFNγR−/− mice at day 7 using a monoclonal antibody against the virus ORF59 (red) and the anti-FSP-1 antibody (green). Merged image shows nuclear localization of viral antigen in FSP-1 positive cells (arrows). Magnification ×400. Staining is representative of 3 different animals. (C) Dual IHC on formalin-fixed paraffin-embedded lung sections of MHV68 infected IFNγR−/− mice at day 120. Epithelial cells expressing mesenchymal cell markers (arrow) were detected using anti-TTF-1 (brown nuclei) and anti-FSP-1 (red cytoplasm) antibodies. Magnification ×400. Box shows area depicted with magnification ×1000. Staining is representative of 5 different animals. (D) Dual immunofluorescence staining in frozen lung sections of MHV68 infected IFNγR−/− mice at day 120 using anti-pro-SP-B (red) and anti-N-cadherin (green) antibodies. Yellow cells indicate type II cells expressing N-cadherin mesenchymal cell marker (arrows). Nuclei were visualized by DAPI staining (blue). Magnification ×200. Staining is representative of 5 different animals.

Previously, we showed that acute MHV68 infection in IFNγR−/− mice was followed by virus reactivation and the development of progressive and multifocal fibrosis [8]. Since EMT is considered one possible source for fibroblasts in pulmonary fibrosis, and since Twist appears to be important for EMT, we explored for the presence of mesenchymal markers in the lung epithelial cells of IFNγR−/− mice infected with MHV68. First, to identify MHV68 infected lung epithelial cells, we performed immunofluorescent staining using an anti-Pro-surfactant C (Pro-SP-C) antibody and a polyclonal anti-MHV68 antibody. The peak of lytic replication of the virus was found at days 7–9 post-infection. At this time, numerous type II alveolar epithelial cells expressing viral antigens were identified in the murine lungs (Fig. 4A). In addition, virus-infected epithelial cells identified by a monoclonal antibody against the viral open reading frame 59, an early DNA replication protein, expressed the mesenchymal cell marker, FSP-1 (Fig. 4B). Thus, early after infection, alveolar epithelial cells show an aberrant pro-fibrotic phenotype.

We then turned our attention to the late time points coinciding with the period of fibrosis. Lung tissues from mock- and MHV68-infected IFNγR−/− mice were harvested after 120 days of infection. Double IHC staining of lung tissue was performed to detect epithelial and mesenchymal markers. Lung tissue of mock infected mice expressed only the epithelial marker, thyroid transcription factor-1 (TTF-1), in the nuclei of alveolar epithelial cells. In contrast, lung specimens from MHV68-infected mice demonstrated nuclear staining for TTF-1 as well as diffuse cytoplasmic staining for FSP-1 in alveolar epithelial cells (Fig. 4C). In parallel experiments tissue was stained with another mesenchymal marker, N-cadherin, and another epithelial marker, Pro-SP-C (Fig. 4D). A semi quantitative analysis of the percentage of single (pro-SP-C) versus double (pro-SP-C + N-cadherin) positively stained cells revealed a range between 7 to 20% of double-labeled cells. Thus, a significant percentage of lung epithelial cells of IFNγR−/− mice infected with MHV68 revealed features of both epithelial and mesenchymal phenotypes consistent with a role for EMT in virus-induced lung fibrosis.

### Expression of Twist and evidence for EMT in lung tissue from IPF patients

**Figure 5:**
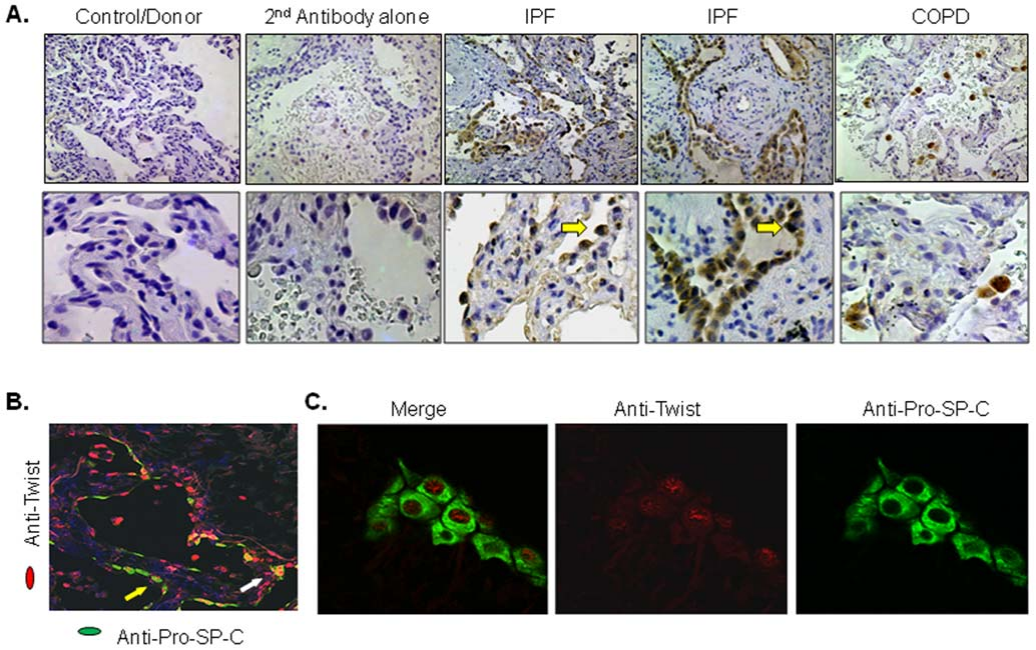
Twist expression and localization in lung tissues of control, COPD and IPF patients. (A) Determination of Twist expression by IHC analysis in sections of control/donor, COPD, and IPF lungs. No Twist expression was detected in control lung. Epithelial cells covering fibrotic areas of IPF lung showed abundant nuclear and cytoplasmic Twist positive staining (arrows). COPD lungs showed strong Twist staining in alveolar macrophages but staining was negative in epithelial cells. Upper row pictures have magnification ×100; lower row – magnification ×400. Stainings are representative of 3 independent assays using at least 3 donors, 1 COPD and 13 IPF lung tissues. All 13 IPF samples demonstrated Twist expression. (B) Dual immunofluorescent staining in frozen lung sections of IPF lung using anti-pro-SP-C (green) and anti-Twist (red) antibodies. Numerous type II cells were found expressing Twist in the nuclei (white arrow) and cytoplasm (yellow arrow). Nuclei were visualized by DAPI staining (blue). Magnification ×200. (C) Confocal microscopic image analyses of dual immunofluorescent staining for Twist (red) and pro-SP-C protein (green). Notice nuclear localization of Twist and cytoplasmic pro-SP-C in the same lung epithelial cells. Magnification ×1000. Stainings are representative of 3 independent assays in lung tissues from 3 IPF patients.

After corroborating the occurrence of EMT in animals with virus-induced lung fibrosis, we then determined the relevance of these findings in humans with IPF. Studies with IPF and control tissue samples were approved by the Emory University Institution Review Board. We collected a total of 13 tissue samples from IPF patients who were undergoing lung transplantation; 3 lung control samples (all donor lung specimens at the time of transplantation) and 1 from a COPD patient. All IPF lung specimens had histopathological diagnosis of usual interstitial pneumonia (UIP). We first analyzed for Twist protein expression and found that Twist immunoreactivity was abundant and localized mostly in the nuclei and cytoplasm of epithelial cells overlying fibrotic areas or honeycombing cysts (Fig. 5A). In normal lung and lung samples from the patient with COPD, cytoplasmic staining for Twist was detected only in alveolar macrophages. Next, we determined the type of cells that expressed Twist by double immunostaining. Analysis of images performed by fluorescent and confocal microscopy revealed nuclear and/or cytoplasmic staining for Twist in 75.3% (±12.3%) of type II alveolar cells in IPF samples as identified by positive staining with anti-pro-SP-C antibody (Fig. 5B, C).

**Figure 6:**
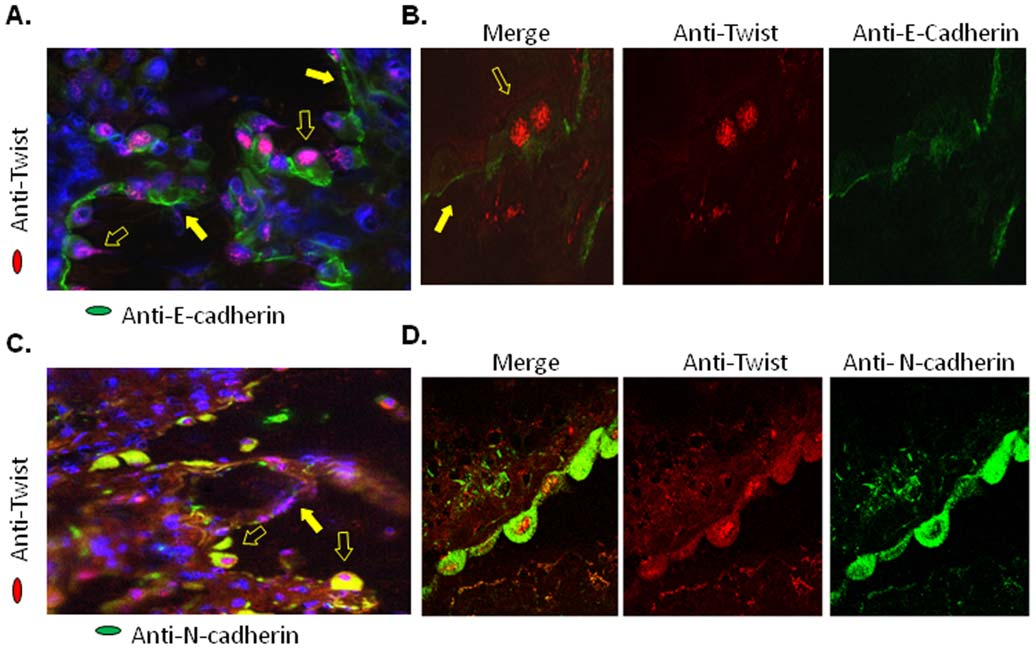
Expression of EMT markers, E-cadherin and N-cadherin, in Twist positive cells in IPF lung tissue. (A) Dual immunofluorescence and (B) confocal microscopic image analyses of frozen lung sections of IPF lung stained with anti-E-cadherin (green) and anti-Twist (red) antibodies. E-cadherin staining was localized in the basolateral membrane in Twist negative cells (closed arrow). Twist positive cells showed weaker E-cadherin staining or cytoplasmic localization (open arrow). Magnification ×400 and ×1000 respectively. (C) Double immunofluorescence and (D) confocal microscropic image analyses of frozen lung sections of IPF lung stained with anti-N-cadherin (green) and anti-Twist (red) antibodies. Notice nuclear Twist positive cells showing N-cadherin (green) positive staining in the cytoplasm (open arrows). Some cells showed Twist positive staining but were negative for N-cadherin (close arrows). Magnification ×200 and ×1000 respectively. Stainings are representative of 3 independent assays in lung tissues from 3 IPF patients.

To determine whether molecular changes associated with EMT could be detected in lung epithelial cells that expressed Twist, we probed for both epithelial and mesenchymal markers. Analysis of fluorescence and confocal microscope images revealed that Twist positive epithelial cells had abnormal distribution of the epithelial marker, E-cadherin, in the cytoplasm. In contrast, epithelial cells without Twist expression retained immunoreactivity for E-cadherin in the normal site that is within the basolateral domains of cell membranes (Figure 6A, B). Further, we investigated whether Twist-positive cells expressed N-cadherin, one of the mesenchymal markers used in EMT studies and a Twist target gene [24], [26], [27], [28]. Double immunofluorescence staining revealed that 95% of cells with strong cytoplasmic immunoreactivity for N-cadherin were also Twist-positive (Fig. 6C). Confocal fluorescence microscopy imaging confirmed co-expression of nuclear Twist and cytoplasmic N-cadherin in alveolar epithelial cells in areas of fibrotic remodeling (Fig. 6D). Taken together, these data show that Twist is strongly expressed in IPF lung tissue and that Twist positive type II alveolar epithelial cells in IPF lung tissue reveal changes associated with EMT.

**Figure 7:**
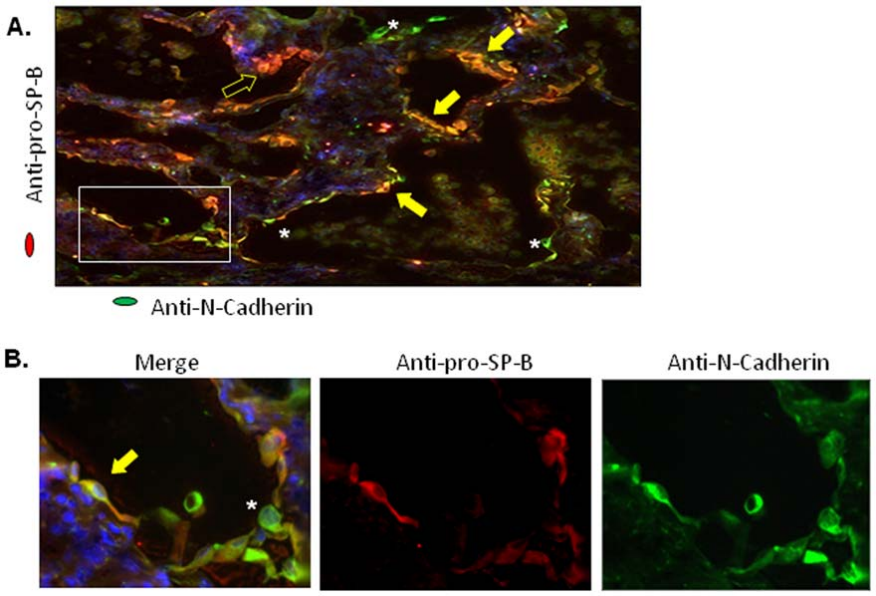
Expression of mesenchymal (N-cadherin) and epithelial (pro-SP-B) markers in IPF lung tissue. (A) Immunofluorescent staining of lung frozen sections of IPF lung using anti-N-cadherin (green) and anti-pro-SP-B (red) antibodies. Type II cells were detected by Pro-SP-B staining (red, open arrow). Yellow cells indicate type II cells expressing the mesenchymal cell marker N-cadherin (closed arrows). Scattered green cells were observed intercalating red and yellow type II epithelial cells (asterisks). Magnification ×100. Box shows area depicted at magnification ×1000 in (B). Nuclei were visualized by DAPI staining (blue). Stainings are representative of 3 independent assays in lung tissues from 3 IPF patients.

To determine the frequency of EMT in IPF lungs, dual immunofluorescence microscopy studies were performed on IPF lung tissue sections using epithelial and mesenchymal markers. As shown in Fig. 7A, IPF lung sections demonstrated heterogeneous staining. Twenty nine percent of the alveolar epithelial cells identified by pro-SP-B positive staining were also positive for N-cadherin. There were alveolar lining cells that expressed only N-cadherin in the cytoplasm (Fig. 7B). These findings suggest that EMT is not a static process, and that mesenchymal transition of epithelial cells is not a synchronized process in the IPF lung.

### Expression of Twist was associated with EBV infection in IPF lung samples

We and others have demonstrated that more than half of IPF patients show evidence of chronic EBV infection in their lungs [1], [2], [4]. Therefore, we asked the question whether EBV infection was associated with Twist expression in these patients. We measured EBV copy number using real time PCR in frozen samples. Nine out of thirteen (69.2%) lung samples from IPF patients were positive for EBV. The range of EBV copy number per million cells was between 6.2 and 18,000. Control lung samples (from donors) and the COPD lung tissue was EBV negative.

**Figure 8:**
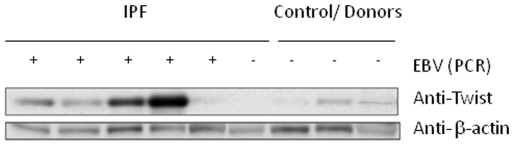
Twist is expressed in IPF lung tissue samples where EBV is detected by Real Time-PCR. Western blot analysis for the expression of Twist was performed in whole cell lysates of control donors and IPF lungs. The blot was stripped and reprobed with anti-β-actin antibody as a loading control. Same lung samples were tested for the presence of EBV genome [(+) ≥10^3^ EBV DNA copies per 10^6^ cells] by real time PCR.

The level of Twist protein in 6 IPF lung samples (5 EBV positive, 1 EBV negative), and 3 EBV negative donor specimen was assessed using Western blot analysis. Twist expression was detected at different levels in all 5 EBV positive IPF samples. In contrast, Twist expression was barely detectable in the EBV negative IPF lung and weakly positive in control (donor) lung samples (Fig. 8).

## Discussion

Although EMT has been implicated in lung fibrosis, the mechanisms underlying EMT remain unclear. In this report, using an *in vitro* and *in vivo* model of MHV68 infection of lung epithelial cells, we show that γ-herpes virus infection induces Twist and expression of mesenchymal markers. Inhibition of Twist expression by shRNA in MLE15 cells infected with MHV68 resulted in persistent E-cadherin expression and lower expression of collagen compared to cells without Twist knockdown. Importantly, ectopic overexpression of Twist in MLE-15 was sufficient to reduce the expression of E-cadherin, a protein required in the cell-cell contact and usually downmodulated during EMT, and to increase the expression of FSP-1, fibronectin and collagen, mesenchymal cell markers. Furthermore, this report is the first to demonstrate upregulation of Twist protein expression in a murine model of virus-induced lung fibrosis and in IPF lung tissue. Twist was expressed in the nuclei of alveolar type II epithelial cells of MHV68 infected lungs and was not expressed in mock-infected lung tissue. The levels of Twist changed with time after lung infection starting with low levels in early infection and high levels at the time of chronic MHV68 infection. Expression of Twist in IPF lungs was upregulated significantly. Furthermore, expression of Twist in alveolar epithelial cells in IPF was associated with expression of pro-SP-C protein and the mesenchymal cell marker N-cadherin. In addition, Twist-positive lung epithelial cells in IPF tissues had cytoplasmic instead of membranous localization of E-cadherin.

Microarray analysis of IPF tissue identified an IPF-specific gene expression signature characterized by the up-regulation of genes involved in tissue remodeling and enriched with genes associated with lung development [29]. Among the developmental genes discovered were members of the Sry-related high mobility group-box and forkhead box families, and genes related to the Wnt/β-catenin pathway. Twist has been shown to be up-regulated in response to Wnt1 expression in mouse mammary epithelial cell lines and tumors [30]. Twist expression is also induced by NF-κB, insulin-like growth factor 1, HIF-1, and EGF/EGFR signaling pathways [31]. Once expressed, Twist directly or indirectly represses E-cadherin expression via E-boxes that are also targeted by Snail and SIP1. However, the repression of E-cadherin is not sufficient to induce EMT and ectopic expression of E-cadherin does not restore the epithelial phenotype in Twist-overexpressing cells [27]. Thus, EMT completion requires the acquisition of mesenchymal markers.

Twist regulates EMT, cell movement, and tissue reorganization during early embryogenesis. Similarly, Twist expression in tumor cells is associated with EMT and increased cell motility, suggesting that Twist may contribute to metastasis. Suppression of Twist expression in highly aggressive 4T1 mammary carcinoma cells specifically inhibits their ability to metastasize from the mammary gland to the lung [27]. Twist has been also related to EMT in a model of renal fibrosis induced by unilateral ureteral obstruction [15]. In that model, Twist expression was increased in tubular epithelia of the dilated tubules and the expanded interstitial areas where proliferating cells were frequently found in a time-dependent manner. The genes downstream of Twist that are essential to initiate and maintain EMT are still under investigation. In breast carcinoma cells, Twist causes transcriptional repression of E-cadherin, α, β and γ-catenins, and stimulates the expression of mesenchymal cell markers like fibronectin, vimentin, α-smooth muscle actin and N-cadherin as well as the angiogenic factor VEGF [27], [32]. Additionally, Twist is able to inhibit oncogene- and p53-dependent cell death and, therefore, considered as an anti-apoptotic factor [33].

Recently, several studies demonstrated EMT induction in the setting of viral infection. Li et al. showed positive expression of hepatitis C virus core protein associated with decreased expression of E-cadherin and α-catenin in conjunction with increased expression of N-cadherin, vimentin, and fibronectin in tissues from cholangiocarcinoma [34]. In a study of cervical cancer, the early stages of keratinocyte transformation by HPV16 were characterized by cellular changes associated with EMT including reduction in expression of cytokeratin, formation of desmosomes, adherents junctions and focal adhesions [35]. As we mentioned before, expression of the EBV protein LMP-1 in nasopharyngeal carcinoma cells was associated with Twist expression and EMT [24]. Virus infection has also been shown to contribute to TGF-β activation and expression of extracellular matrix components in endothelial cells. Infection of endothelial cells with cytomegalovirus (CMV) upregulated integrin-β6 followed by activation of the TGF-β pathway and expression of collagen IV [36]. Those observations are intriguing in view that EMT in alveolar epithelial cells is induced by linking TGF-β and β-catenin signaling through integrins [13], [37].

Strong evidence for EMT *in vivo* in the setting of epithelial injury/stress is difficult to obtain because of the isolated and possible transient nature of the process; such transitioning cells are difficult to track. However recent studies with bone marrow chimeras and transgenic reporter mice showed that during renal fibrogenesis fibroblasts are derived both from bone marrow and by local EMT with EMT being the dominant source of the fibroblasts [38]. EMT has also been detected in IPF lung tissue and animal models of pulmonary fibrosis [12], [13]. Using a triple transgenic mouse reporter system, Kim et al. demonstrated that EMT plays an important role during lung fibrogenesis and may be more widespread than previously thought [13].

Our data suggest that Twist might contribute to the EMT process in pulmonary fibrosis. Furthermore, they suggest that γ-herpes virus might be, in some IPF cases, the source of injury precipitating EMT. We demonstrated that biopsies with relatively high Twist expression were positive for EBV genome. Several other studies suggest a potential role for EBV in viral epithelial injury and potential EMT development. In a viral cell line model, TGFβ1 was shown to be induced in epithelial cells following EBV lytic phase induction. TGFβ1 expression was promoted by the EBV early genes Rta and Zta [39]. In human nasopharyngeal carcinoma tissue, expression of Twist and EBV LMP-1 was directly correlated. EBV LMP-1 caused morphologic and molecular changes of EMT in cultured epithelial cells and this EMT was reversed by suppressing Twist [24].

Although our studies suggest that virus-induced Twist activation might represent an important mechanism for EMT in IPF, further studies will be needed to test the true role of Twist in EMT *in vivo* and to determine the role of virus infection in the fibrogenesis pathways that characterize IPF and related lung fibrotic disorders.

In summary, our studies, suggest that EMT may be a cellular mechanism of fibrogenesis in the lung associated with virus-induced epithelial injury. Twist is a well known master and a transcriptional regulator of EMT during embryogenesis and metastasis. The abundant expression of Twist in alveolar epithelial cells is likely to contribute to EMT and an important source of fibroblasts in IPF lungs. The identification of the downstream effector pathways that are activated during EMT holds the promise of revealing new diagnostic markers of early stages of pulmonary fibrosis and, quite possibly, novel targets for anti-fibrotic therapeutics.

## Materials and Methods

### Antibodies and reagents

Antibodies to detect MHV68, polyclonal anti-MHV68 and chicken monoclonal anti-MHV68 ORF59, were a generous gift from Dr. Samuel Speck (Department of Microbiology/Immunology, Emory University) [8], [40]. Polyclonal rabbit anti-human pro-SP-C (Chemicon International); mouse monoclonal anti-human pro-SP-B (Thermo Fisher Scientific); rabbit polyclonal anti-S100A4 (anti-FSP-1) (Dako Cytomation); mouse monoclonal anti-TTF-1 (Novocastra); rabbit polyclonal anti-Twist (sc-15393, Santa Cruz Biotechnology, Inc); mouse monoclonal anti-Twist (sc-81417, Santa Cruz Biotechnology, Inc); mouse monoclonal anti-βactin (Sigma-Aldrich, Saint Louis, MO); rabbit polyclonal anti-E-cadherin (Santa Cruz Biotechnology, Inc); rabbit polyclonal anti-N-cadherin (Abcam); rabbit polyclonal anti-Collagen I (Millipore); rabbit polyclonal anti-Collagen IV (Santa Cruz Biotech Inc); goat polyclonal anti-Vimentin (Chemicon International); rabbit polyclonal anti-Occludin (Zymed Laboratories); rabbit polyclonal anti-Fibronectin (Sigma) antibodies for immunofluorescence and immunoblot assays were used according to the manufacturer recommendations.

### Cell culture and treatment

MLE-15 cells were obtained from Dr. Brigham Willis (University of Texas) and were maintained in HITES medium (RPMI 1640 (GIBCO, Gaithersburg, MD) supplemented with 2% fetal bovine serum (Thermo Scientific HyClone), 100 U/ml penicillin, 100 µg/ml streptomycin, 1% insulin-transferrin-sodium selenite, 5 µg/ml transferrin, 10 nM hydrocortisone, 10 nM β-estradiol, 2 mM glutamine, and 2 mM HEPES).

### Animals and animal infection

The animal experiments were reviewed and approved by the Emory University Institutional Animal Care and Use Committee before the experiments were conducted. Animals were infected as described before [8], [9]. Briefly, IFNγR−/− mice were inoculated intranasally with 1×10^5^ plaque forming units (pfu) of MHV68 mixed in Dulbecco modified Eagle medium (DMEM) supplemented with 10% fetal calf serum to a total volume of 40 µl. Inoculation of supernatant from homogenates of uninfected NIH3T12 cells was used as a mock infected control.

### Human tissues

Lung tissues were obtained from 13 IPF patients with histological evidence of UIP, and from 4 control subjects (organ donors, COPD). Samples were immediately frozen for double immunofluorescence staining or placed in 4% (w/v) paraformaldehyde for IHC. The study protocol was approved by the Emory University IRB (protocol 2005–361). Informed consent was obtained in written form from each subject.

### MHV68 in vitro infection

MLE15 epithelial cells (7×10^4^ cells/well in 4-well chamber slides), were infected with MHV68 with a multiplicity of infection (MOI) of 0.01, fixed 48 hours post-infection with 4% paraformaldehyde (Sigma-Aldrich), and permeabilized in 0.02% Triton X-100 (Sigma-Aldrich). Similarly, cells were infected in 6 well plates and whole cell extracts were prepared.

### Twist transfection assay

MLE15 cells were transfected by electroporation with Twist cDNA (plko.1 vector, Origene) and pcNeo DNA in a 5∶1 ratio. After electroporation, cells were washed and cultured with HITES medium. Positive selection was done using G418 (400 µg/ml) after the second day of transfection.

### Lentivirus transduction

MLE-15 cells were transduced with lentivirus expressing mouse Twist1 shRNA and non-target control shRNA (MISSION Sigma-Aldrich) at an MOI of 2. After 24 h, cells were infected with MHV68 at an MOI of 0.05. The cells were washed with PBS and harvested for protein isolation at 48 hours post-MHV68 infection.

### Quantitative real-time PCR

Real time PCR reactions were Taqman-based (Qiagen, Valencia, CA). DNA extractions were carried out using the Qiagen DNeasy Blood & Tissue Kit (Valencia, CA). EBV PCR targeted the BamH1W viral genome segment and was performed as described before using frozen human lung tissue [41]. RNAseP was used as housekeeping gene.

### Immunohistochemistry

Human and murine lungs were placed in 4% paraformaldehyde and processed for paraffin embedding. Sections (5 µm) were cut, mounted on the slides, subjected to the antigen retrieval in a decloaking chamber (BioCare medical). Endogenous peroxidase activity was quenched with 3% peroxide for 5 min. Immune complexes were visualized with biotinylated secondary antibody and 3,3′-diaminobenzidine tetrahydrochloride using streptavidin-biotin complex method. Double immunohistochemistry was carried out using EnVision G//2 doublestain system, Rabbit/Mouse (DAB+/Permanent Red) kit (DAKO Cytomation, Denmark).

### Immunofluorescence assay

Frozen human and murine lungs were cut (5 µm) and fixed in acetone/methanol (1∶1). After washing in several changes of PBS, tissue sections on glass slides and MLE15 cells on chamber slides were incubated with primary antibodies in antibody diluent with background reducing component (Dako Cytomation, Denmark) overnight at 4°C. The indirect immunofluorescence assay was performed by incubation with secondary antibodies conjugated to Alexa Fluor 594 and/or Oregon Green 488 (Invitrogen). Nuclei were visualized by 4,6-diamidino-2-phenylindole staining (DAPI, Sigma-Aldrich, Saint Louis, MO). Slides were analyzed under fluorescent microscope Olympus BX4 equipped with Olympus SN 1H045294-H camera and Zeiss Axioplan 2 imaging LSM 510 META confocal microscope. Semi-quantitative evaluation of single positive cells (pro-SP-C) and double positive cells (pro-SP-C+N-cadherin) was perfomed using the Axiovision morphometric software. Five representative pictures at 40× magnification were taken from the left lung of mice at day 120 post-infection (n = 5) and cells were counted. Pictures were taken from areas without big airways or blood vessels.

### Western blot analysis

MLE15 cells and human and murine lung tissue specimens were homogenized in extraction buffer (50 mM HEPES, 250 mM NaCl, 5 mM EDTA, 0.1% NP-40, 1 mM PMSF,1 mM DTT supplemented with Protease inhibitor cocktail; BD Biosciences, San Diego, CA). Whole proteins were extracted by centrifugation (12,000x g) for 10 min at 4°C. Samples containing 40 µg of protein were separated by electrophoresis on SDS 10–20% gradient polyacrylamide gels. Proteins were transferred to PVDF membrane (Millipore, Bedford, MA). Membrane was blocked in 5% skim dry milk and probed overnight at 4°C in TBS-Tween buffer (0.02 M Tris [pH 7.6], 0.1 M sodium chloride, 0.05% Tween 20) with primary antibodies. Membranes were washed in TBS-Tween buffer and incubated with secondary antibodies for 1 h at RT. After washing membranes in TBS-Tween buffer again, they were visualized with SuperSignal West Pico Chemiluminescent Substrate (Pierce Biotechnology, Rockford, IL).
